# MURC/cavin-4 Is Co-Expressed with Caveolin-3 in Rhabdomyosarcoma Tumors and Its Silencing Prevents Myogenic Differentiation in the Human Embryonal RD Cell Line

**DOI:** 10.1371/journal.pone.0130287

**Published:** 2015-06-18

**Authors:** Fiorella Faggi, Silvia Codenotti, Pietro Luigi Poliani, Manuela Cominelli, Nicola Chiarelli, Marina Colombi, Marika Vezzoli, Eugenio Monti, Federica Bono, Giovanni Tulipano, Chiara Fiorentini, Alessandra Zanola, Harriet P. Lo, Robert G. Parton, Charles Keller, Alessandro Fanzani

**Affiliations:** 1 Department of Molecular and Translational Medicine, University of Brescia, Viale Europa 11, 25123, Brescia, Italy; 2 Interuniversity Institute of Myology (IIM), Rome, Italy; 3 Institute for Molecular Bioscience, University of Queensland, Brisbane, Queensland 4072, Australia; 4 Department of Pediatrics, Oregon Health & Science University, Portland, OR, United States of America; 5 Children’s Cancer Therapy Development Institute, Fort Collins, CO, United States of America; Ospedale Pediatrico Bambino Gesu', ITALY

## Abstract

The purpose of this study was to investigate whether MURC/cavin-4, a plasma membrane and Z-line associated protein exhibiting an overlapping distribution with Caveolin-3 (Cav-3) in heart and muscle tissues, may be expressed and play a role in rhabdomyosarcoma (RMS), an aggressive myogenic tumor affecting childhood. We found MURC/cavin-4 to be expressed, often concurrently with Cav-3, in mouse and human RMS, as demonstrated through *in silico* analysis of gene datasets and immunohistochemical analysis of tumor samples. *In vitro* expression studies carried out using human cell lines and primary mouse tumor cultures showed that expression levels of both MURC/cavin-4 and Cav-3, while being low or undetectable during cell proliferation, became robustly increased during myogenic differentiation, as detected via semi-quantitative RT-PCR and immunoblotting analysis. Furthermore, confocal microscopy analysis performed on human RD and RH30 cell lines confirmed that MURC/cavin-4 mostly marks differentiated cell elements, colocalizing at the cell surface with Cav-3 and labeling myosin heavy chain (MHC) expressing cells. Finally, MURC/cavin-4 silencing prevented the differentiation in the RD cell line, leading to morphological cell impairment characterized by depletion of myogenin, Cav-3 and MHC protein levels. Overall, our data suggest that MURC/cavin-4, especially in combination with Cav-3, may play a consistent role in the differentiation process of RMS.

## Introduction

Rhabdomyosarcoma (RMS) is a myogenic tumor classified as the most common soft-tissue malignancy of childhood [1–3]. Despite the expression of proteins required for myogenesis, such as the bHLH (basic helix-loop-helix) transcription factors myogenin and MyoD (myogenic differentiation protein) [4–6], RMS cells fail to complete myogenic differentiation [7]. Histopathological criteria define two predominant subtypes referred to as embryonal (eRMS) and alveolar (aRMS), accounting for about 60% and 25% of all RMS, respectively [8]. While eRMS is composed of spindle-shaped or round cells resembling embryonic skeletal muscle, aRMS is formed by aggregates of small round undifferentiated cells separated by dense hyalinized fibrous septa reminiscent of lung alveolar architecture. Patients who have localized RMS have a 5-year survival greater than 70% following a multimodal approach that includes chemotherapy, radiation therapy, and surgery; yet, overall survival of patients with metastasis remains poor [9, 10]. The genomic landscape causative of eRMS is characterized by a number of genetic aberrations, including the loss of heterozygosity at 11p15.5 responsible of IGF-2 (insulin-like growth factor 2) overexpression [11, 12], gain of chromosomes [13, 14], somatic mutations in cell cycle genes (i.e., *CTNNB1*, *FBXW7* and *BCOR*) [15], and in several tyrosine kinase genes (i.e., *PDGFRA*, *ERBB2*, *FGFR4*) [16] and transducers (i.e., *NRAS*, *KRAS*, *HRAS*, *PIK3CA*, *CTNNB1*) [17, 18] that lead to deliberate activation of tyrosin kinase receptors /RAS (rat sarcoma viral oncogene) /PI3K (phosphoinositide 3-kinase) axis [15]. In addition, defects in the p53 pathway [19], Sonic Hedgehog signaling [20–23] and sarcomeric proteins involved in muscular contraction and integrity (i.e., dystrophin, alpha-sarcoglycan and dysferlin) have been reported to favor eRMS formation [24–29]. Conversely, aRMS is dominated by a t(2;13)(q35;q14) chromosomal translocation that juxtaposes the DNA binding domains of the *PAX3* (paired box 3) gene in frame with the partial DNA binding domain and full transactivation domain of the *FOXO1* (forkhead box O1) gene, resulting in the expression of the fused Pax3-Foxo1 transcription factor [30]. This factor drives transcription of numerous Pax-3 downstream genes in a deliberate manner, contributing to suppress apoptosis and differentiation processes [31, 32] and conferring resistance to stress conditions such as irradiation *in vitro* and *in vivo* [33]. To date, the presence of a *PAX3–FOXO1* gene fusion is a strong indicator of poor prognosis as fusion-negative aRMS have better resolution mimicking the clinical course of eRMS in the majority of patients [34, 35].

Caveolins (i.e., Cav-1,-2,-3) [36, 37] and Cavins (i.e., Cavin-1,-2,-3,-4) [38–43] are family proteins that cooperate in the biogenesis and function of *caveolae*, specialized invaginations of the plasma membrane involved in a variety of cellular processes, including endocytosis, lipid homeostasis and intracellular signalling [44, 45]. Lack or improper function in some of these protein members have been reported to affect caveolar function, hence disturbing the whole body homeostasis and contributing significantly in the onset and/or progression of diseases like diabetes, muscular dystrophies and cancer [44, 45]. We [46–48] and others [49] have previously documented the expression of Caveolins in RMS and more recently shown the important contribute of Cav-1 and Cavin-1 on tumor growth [48, 50]. In this work we have investigated whether Muscle-Restricted Coiled-coil (MURC)/cavin-4, a plasma membrane and Z-line associated protein exhibiting an overlapping distribution with Cav-3 in heart and muscle tissues [51, 52], may be expressed and play a role in RMS. For this purpose, we have used an *in silico* approach combined with the immunohistochemical analysis of tumor samples. In addition, we have investigated MURC/cavin-4 expression *in vitro* by means of human cell lines and mouse primary tumor cultures established from conditional transgenic mice [53, 54]. Finally, the effects of *MURC/CAVIN-4* gene knock-down on the proliferation and differentiation of human embryonal RD cell line have been evaluated.

## Materials and Methods

All reagents were from Sigma-Aldrich (Milan, Italy), unless otherwise stated. Cell culture materials were purchased from Jet-Biofil (Carlo Erba Reagents-Dasit Group, Cornaredo, Milan, Italy).

### Microarray gene expression data analysis

All analyses of microarray gene expression data were performed with the Partek Genomics Suite software version 6.6 (Partek, St. Louis, MO, USA) and R software 3.02 (free version). Briefly, the microarray raw dataset with the accession number GSE22520 [54], deposited in the NCBI Gene Expression Omnibus database, were reprocessed by the background correction, normalization and summarization of probe intensities using the robust multiarray average analysis to determine the specific hybridizing signal for each probe set. The ILMN_1228951, ILMN_2603299 and ILMN_1241214 probes were representative of *MURC/CAVIN-4*, *CAV-3* and *MHC* transcript, respectively. After background correction, the data expression were corrected for perfect match intensity and were transformed in base-2 logarithm [55]. Quality control was performed by investigating principal component analysis to detect grouping patterns in the samples and identify the outliers, as well as for evaluating whether batch effect significantly affected the data. To detect if each gene was differentially expressed between mouse aRMS/eRMS *vs* skeletal muscle samples, we analyzed the median differences using a Kruskal—Wallis test. To visualize gene clustering, we employed the heat map analysis. A heat map is a graphical representation of the data where the individual values contained in a matrix are represented with colors. The method displays the genes on the *x*-axis and the 21 samples on the *y*-axis, adding also two dendrograms which are the output of two hierarchical cluster analyses computed on genes and samples, respectively. In detail, rows and columns of the data matrix are reordered based on row and column means; in this way similar values are placed near each other according to the clustering algorithm [56]. Data are standardized then allowing comparisons among potentially very different scale values. *p-values* < 0,05 were used as criteria to evaluate significant difference in gene expression.

### Antibodies

The following primary antibodies were used: rabbit polyclonal anti-MURC/cavin-4 for immunohistochemical analysis (Code: HPA020973, Sigma-Aldrich, Milan, Italy); goat polyclonal anti-MURC/cavin-4 for immunoblotting analysis (Code: SC-163021, Santa Cruz Biotechnology, Dallas, TX, USA); rabbit polyclonal anti-MURC/cavin-4 for immunofluorescence analysis (as described in [52]); mouse monoclonal anti-Cav-3 (Code: 610420, BD Transduction Laboratories, Buccinasco, Milan, Italy); rabbit anti-myogenin (Santa Cruz Biotechnology, Dallas, TX, USA, 1:500 dilution); mouse monoclonal anti-MHC (Code: SC-32732, Santa Cruz Biotechnology, Dallas, TX, USA); mouse monoclonal anti-total and—phosphorylated ERK1/2 (extracellular regulated kinase 1/2) (Tyr204) (Code: SC-135900 and SC-7383, Santa Cruz Biotechnology, Dallas, TX, USA); mouse monoclonal anti-GAPDH (glyceraldehyde 3-phosphate dehydrogenase) (Code: MAB374, Millipore, Darmstadt, Germany) and mouse monoclonal anti-alpha-tubulin (Code: T5168, Sigma-Aldrich, Milan, Italy).

#### Immunohistochemical analysis

Mouse tumor samples were established from transgenic mouse models at the Oregon Health & Science University, in accordance with the Guidelines for the Care and Use of Laboratory Animals, following approval by the Institutional Animal Care and Use Committee (IACUC) at the University of Texas Health Science Center at San Antonio or Oregon Health and Science University. Every effort was made to minimize suffering in tumour bearing animals, as described in [54]. Formalin-fixed paraffin embedded tissue samples from patients who underwent surgery were retrieved from the archive of the Department of Pathology, Spedali Civili of Brescia, in agreement with protocols approved by the Institutional Review Board (IRC), Spedali Civili (Brescia, Italy), and upon written informed consent from the patient. Sections of 2 micron were cut from paraffin embedded blocks and subjected to immunohistochemical (IHC) analysis. Briefly, sections were de-waxed, re-hydrated and endogenous peroxidase activity blocked by 0.3% H_2_O_2_/methanol for 20 minutes. Heat-induced antigen retrieval was performed using a microwave-oven in 1 mM Citrate buffer (pH 6.0). Sections were then washed in Tris Buffered Saline (TBS) (pH 7.4) and incubated at 37°C overnight in TBS/1% bovine serum albumin (BSA) with the specific primary antibody. Single immunostain has been revealed by CheMATE En Vision HRP Labelled Polymer system (DAKO, Milan, Italy) or NovoLinkTM Polymer Detection System (NovocastraTM laboratories Ltd, Milan, Italy) followed by diaminobenzydine as chromogen and Haematoxylin as counterstain. For double immunostains, after completing the first immune reaction, the second primary antibody has been applied and labelled using MACH 4TM Universal AP Polymer Kit (Biocare Medical, Milan, Italy); chromogen reaction was developed with Ferangi BlueTM Chromogen System (Biocare Medical, Milan, Italy) and nuclei were counterstained with Methyl Green. Images have been acquired by Olympus DP70 camera mounted on Olympus Bx60 microscope, using CellF imaging software (Soft Imaging System GmbH, Berlin, Germany).

#### Cell cultures and drug treatments

*In vitro* studies were conducted using the human embryonal RD cells, RD12, RD18 and alveolar RH30 cells, as described in [50]; the primary mouse embryonal U57810 and alveolar U23674 cultures were derived from transgenic mice in which either p53 loss or concomitant p53 loss and Pax3-Foxo1 knock-in was restricted to Myf6 (myogenic factor 6)-positive myoblasts, respectively [53, 54]. Cells were routinely maintained under standard conditions (37°C and 5% CO_2_ in humidified incubator) in a growth medium (GM), consisting of high-glucose Dulbecco’s Modified Eagle’s Medium (DMEM) supplemented with 10% fetal bovine serum (FBS) and 100 μg/ml penicillin—streptomycin antibiotics; RH30 cells also received 1% L-Glutamine. To induce myodifferentiation, all the cell lines reaching the confluence received a daily renewed differentiating medium (DM), consisting of DMEM supplemented with 2% horse serum; alternatively, embryonal cells received every day fresh DM added with the chemical PD098059 (10 μM, dissolved in dimethylsulfoxide vehicle, DMSO), a synthetic upstream inhibitor of the RAS/ERK cascade that enhances differentiation [57].

### Generation of MURC/cavin-4 silenced clones

We stably transfected human RD cells with either four different GFP-tagged short hairpin RNAs tailored to *MURC/CAVIN-4* sequence (shMURC) or one random OFF-target sequence as a negative control (shOFF), using Transit-LT1 reagent (Tema-Ricerca, Castenaso, Milan, Italy) according to the manufacturer's protocol. The following shRNA sequences cloned in pGFP-V-RS constructs (Tema-Ricerca/ORIGENE, Castenaso, Milan, Italy) were used (superscript numbers indicate the recognized nucleotides over the MURC sequence): shMURC ^103–131^ (clone TG315318A—GI361266, 5′-TCCACCAGAATCGCCTGTCGAGTGTTACA-3′); shMURC ^901–929^ (clone TG315318B—GI361267, 5′-ACCGAACAGTGGCTGAAGGTGAGGAATGT-3′); shMURC ^178–206^ (clone TG315318C—GI361268, 5′-ACAAAGTAGCCTCCATCGTGGACAGTGTG-3′); shMURC ^366–394^ (clone TG315318C—GI361269, 5′-TATTCCAGGAGAAGTTTCGGTGTCCGACA-3′); shOFF (clone TR30013, 5′-CTTCAAGACCACATACAGATCCAAGAAAC-3′). After antibiotic selection, the GFP staining labeled near the totality of cells and the experiments raised similar results in all the selected clones.

### Giemsa and crystal violet staining

We stained the cells with Giemsa reactive to visualize the presence of elongated myotube-like structures that are indicative of myogenic differentiation. To this end, cells were washed in phosphate buffered saline (PBS) and fixed in methanol at -20°C. Cells were given Giemsa reactive for 4 hours, then were washed three times in PBS. We employed the crystal violet assay to measure cell proliferation. To this end, cells were seeded in 24-well plates at a density of 10x10^3^ and fixed in PFA after 24, 48 and 72 hours in GM. Cells were stained for 10 min with crystal violet solution (0.2% in PBS with 20% methanol) and then collected in 600 μl of SDS solution (1% in PBS). Absorbance of the samples was measured by reading the plate at 540 nm emission wavelengths and was proportional to the amount of proliferating cells that incorporated the crystal violet.

### RNA isolation and semi-quantitative RT-PCR analysis

Total RNA was isolated using a Tri-reagent kit and treated with DNA-free DNase (Promega, Madison, WI, USA). RNA (2 μg) was reverse-transcribed in the presence of 400 Units of Moloney murine leukemia virus reverse transcriptase (MMLV-RT) enzyme (Promega, Madison, WI, USA) and the obtained cDNA template was used for PCR analysis using specific forward and reverse primers (250 nM). In particular, a 560 bp-long human *MURC/CAVIN-4* fragment was amplified with 5′-ATGAAGACCAAGACGCTGC-3′ and 5′-ATGTGCTCCTTGCCTGACTT-3′ primers, a 558 bp-long mouse *MURC/CAVIN-4* fragment with 5′- AATGCTGATAAAATCCACCAGAA-3′ and 5′-ATGTGCTCCTTGCCTGACTT-3′ primers and a 267 bp-long *GAPDH* fragment (complementary to both human and mouse forms) with 5′-CGTGGAGTCTACTGGCGTCTTC-3′ and 5′-GGGAGTTGTCATATTTCTCGTGGTT-3′ primers.

#### Immunoblotting analysis

Protein concentration was calculated by Bradford reagent assay. Equal amounts of protein samples were separated by SDS-PAGE under reducing conditions and transferred to polyvinylidine fluoride (PVDF) membranes. Incubation with specific primary antibodies was followed by horseradish peroxidase-conjugated secondary antibodies (1:4,000 dilution), including donkey anti-goat (Santa Cruz Biotecnology, Dallas, TX, USA), goat anti-mouse IgG (Santa Cruz Biotechnology, Dallas, TX, USA) and donkey anti-rabbit IgG (Thermo Scientific-Pierce, Erembodegem, Belgium). The resulting immunocomplexes were visualized using enhanced chemiluminescence reagent (GeneSpin, Milan, Italy). Immunoreactive bands were quantified using densitometric analyses (Software Gel Pro Analyzer, version 4, MediaCybernetics Inc, Rockville, MD, USA). For detection of MURC/cavin-4, myogenin, MHC, tubulin and ERK1/2 (total and-phosphorylated on Tyr204), protein homogenates were prepared by harvesting cells in cold RIPA lysis buffer, composed by 20 mM Tris-HCl (pH 7.6), 1% Nonidet P40, 0.5% sodium deoxycholate, 0.1% SDS, 50 mM NaCl, and a cocktail of protease inhibitors (Roche, Monza, Monza Brianza, Italy) plus phosphatase inhibitors (1 mM Na_3_VO_4_ and 4 mM NaF). Total homogenates were then centrifuged at 12,000 x g for 10 minutes at 4°C. Triton-insoluble membranous fractions were used for detection of Cav-3 and were obtained by harvesting the cells in cold Triton buffer, composed by 10 mM Tris-HCl (pH 8.0), 1% Triton X-100, 5 mM EDTA, 150 mM NaCl, and a cocktail of protease inhibitors plus phosphatase inhibitors, followed by centrifugation (15,000 x g for 15 minutes at 4°C). Soluble- and detergent-insoluble fractions were obtained by ultra-centrifugation (100,000 x g for 1 hour at 4°C) of total lysates obtained from cells harvested in cold Triton buffer.

### Immunofluorescence analysis

Cells were cultured onto 12 mm glass coverslips coated with FBS and fixed with paraformaldehyde (PFA) for 15 minutes at room temperature (RT); PFA-fixed cells were then permeabilized in 0.1% saponin in PBS for 10 minutes before subsequent incubation in 50 mM NH_4_Cl in PBS for 10 minutes and in blocking solution (composed of 0.2% BSA and 0.2% fish skin gelatin in PBS) for 20 minutes at RT. A 30-minutes incubation in blocking solution containing primary antibodies was followed by 4×5 minutes PBS washes before incubation in blocking solution containing fluorescently tagged secondary antibodies for 20 minutes. After further 4×5 minutes PBS washes, the cells were incubated with DAPI for 1 minute just before rinsing in water and then mounted in Mowiol. Image acquisition of cells, indirectly labeled with Alexa fluorophores (Invitrogen, Life Technologies, Monza, Monza Brianza, Italy), was performed at RT in Acqua-Poly Mount medium (Polysciences, Warrington, PA, USA) using a confocal microscope with photomultiplier tube detectors (LSM 510 Meta; Carl Zeiss, Oberkochen, Germany) equipped with a 63x oil immersion objective (Carl Zeiss Inc., Oberkochen, Germany). The data were captured using the LSM 510 Meta software (Carl Zeiss, Oberkochen, Germany) and unprocessed images were assembled using Photoshop (CS3; Adobe).

### Statistical analysis

The differences between the groups were analyzed by unpaired Student's t tests and One-Way ANOVA test (with Dunnet's post-test), using Prism 4 software for Windows (GraphPad Software, San Diego, CA, USA). Statements of significance were based on a *p-value* of less than 0.05.

## Results

### *In silico* analysis predicts correlation between MURC/cavin-4 and Cav-3 in RMS tumors

**Fig 1:**
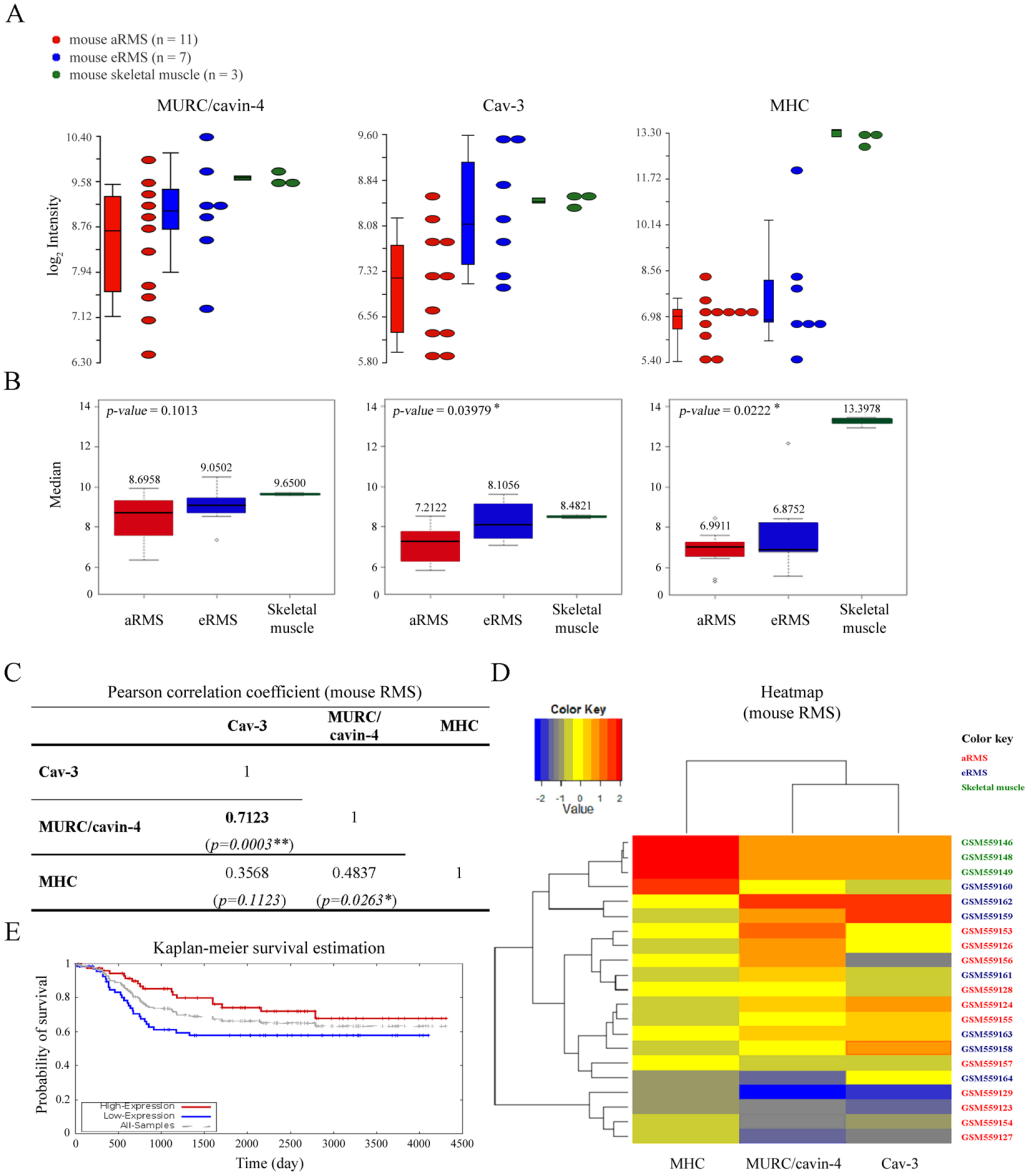
*In silico* analysis. A) Dot-plots and bar charts representative of the transcript levels of *MURC/CAVIN-4*, *CAV-3* and *MHC* genes in aRMS (n = 11) and eRMS (n = 7) *vs* skeletal muscle (n = 3) samples, as calculated after *in silico* analysis of microarray data. In the plot, each dot is a sample of the original data. The Y-axis represents the log_2_ normalized intensity of the gene and the X-axis represents the different types of samples. Bars represent the average ± standard error of the mean. B) *MURC/CAVIN-4*, *CAV-3* and *MHC* transcript levels were represented with box-plots. *MURC/CAVIN-4* gene was not differentially expressed between mouse aRMS and eRMS in comparison to skeletal muscle samples, as determined by Kruskal-Wallis test. *p-values* < 0,05 were used as criteria to evaluate significant difference in gene expression. In each box-plot the median value (black line in the box) is reported in correspondence of every subgroups. C) Heat map analysis on *MURC/CAVIN-4*, *CAV-3* and *MHC* transcript levels in aRMS, eRMS and skeletal muscle samples. Low values of the gene expression are represented with blue, mean values are represented with yellow while high values are represented in red. D) Kaplan-Meier analysis was performed using microarray data from 139 primary human samples [54]. The overall survival in RMS patients with respect to Cav-3 expression is indicated with red and blue curve, respectively.

To analyze the transcriptional levels of *MURC/CAVIN-4*, *CAV-3* and *MHC* in RMS, we employed an *in silico* approach using microarray data available in the NCBI Gene Expression Omnibus database with the accession number GSE22520, which were previously generated by the analysis of primary tumors established from mouse models [54]. By evaluating the variability in the expression levels of *MURC/CAVIN-4*, *CAV-3* and *MHC*, as detected in aRMS (n = 11) and eRMS (n = 7) tumors and skeletal muscle samples (n = 3) (Fig 1A), we found that the medians of *CAV-3* and *MHC* in three groups were statistically different with a *p-value* of 0.0398 and 0.0222, respectively, while the levels of *MURC/CAVIN-4* in tumor samples did not significantly differ from those observed in skeletal muscle (*p-value* = 0.1013) (Fig 1B). Furthermore, we evaluated by means of the Pearson correlation coefficient (*ρ)* that *MURC/CAVIN-4* and *CAV-3* have a high positive linear relationship (*ρ* = 0.7123) that is statistical significant (*p-value* = 0.0003) (Fig 1C), meaning that *CAV-3* and *MURC/CAVIN-4* concurrently increased or decreased in the samples analyzed. To better visualize the gene expression levels we then employed the heat map method (Fig 1D). Consistent with the correlation coefficient, heat map representation confirmed that *MURC/CAVIN-4* and *CAV-3* genes were clustered together, showing a similar expression in aRMS, eRMS and skeletal muscle samples; on the other hand, *MHC* had an independent behavior, reaching higher values in correspondence to skeletal muscle samples, while being at lower values in tumor samples. Interestingly, using data source relative to analysis of 139 primary human tumors [58] (available at the Oncogenomics databases: https://pob.abcc.ncifcrf.gov/cgi-bin/JK), we found that *CAV-3* expression levels correlated with higher probability of patient’s survival, as obtained by means of the Kaplan-Meier survival estimation (Fig 1E); In particular, the three-year overall survival estimated in tumors with high *vs* low *CAV-3* expression was about 85% and 60%, respectively (Fig 1E), suggesting that tumors with higher *CAV-3* may have a more positive prognosis. Unfortunately, it was not possible to perform the same analysis for *MURC/CAVIN-4* because the probe was absent in the microarray.

### MURC/cavin-4 expression frequently matches that of Cav-3 in RMS tumors

**Fig 2:**
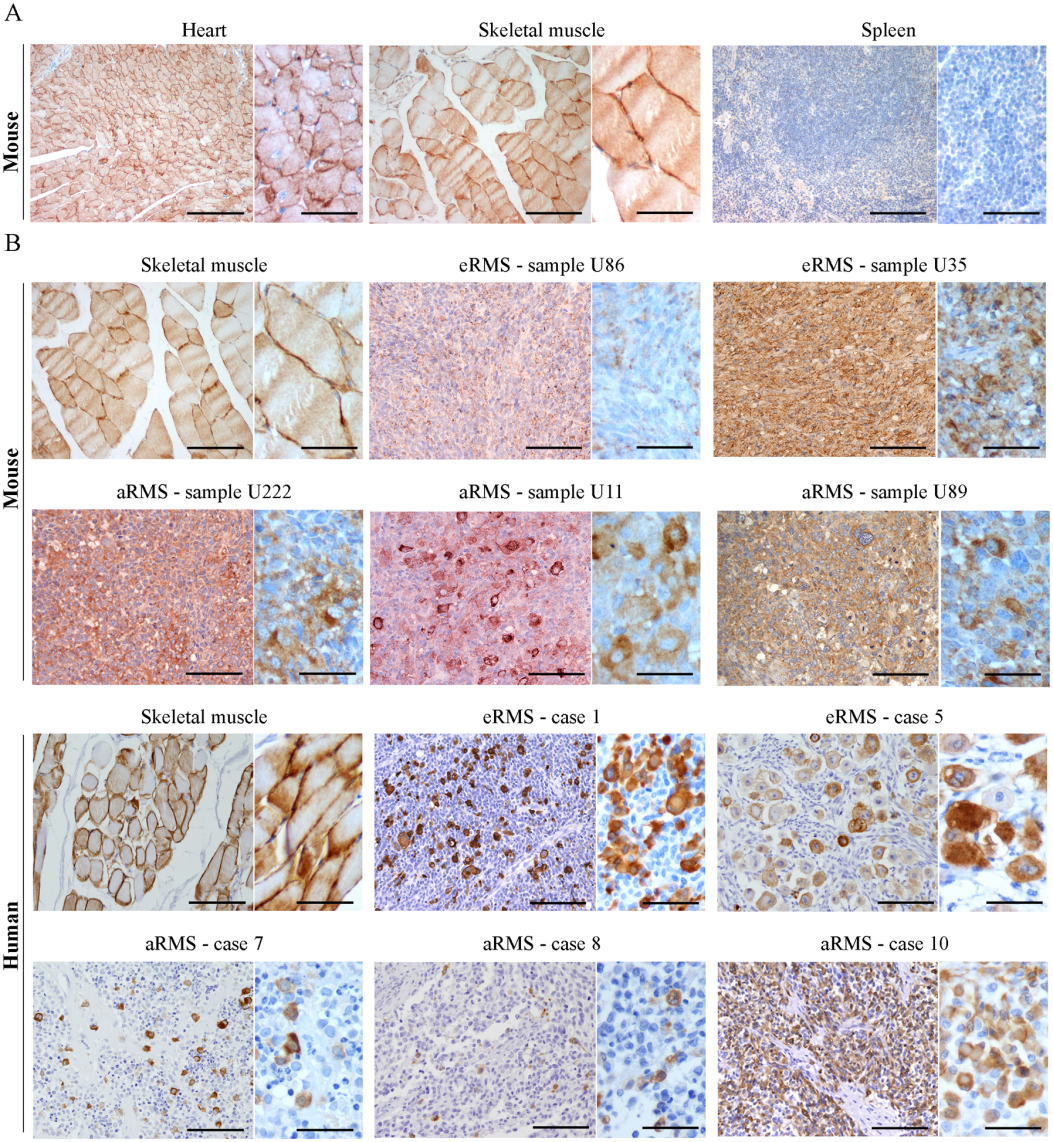
Expression of MURC/cavin-4 in RMS tumors. A) The specificity of MURC/cavin-4 antibody was tested by IHC analysis using mouse tissue samples derived from heart, skeletal muscle and spleen. The latter was expectedly negative to MURC/cavin-4 staining (brown). Images were taken at 20x and 60x magnification. Scale bars:100 μm. B) MURC/cavin-4 staining (brown) was evaluated by IHC analysis on mouse and human tumors (as reported in Table 1). Skeletal muscle served as a positive control. Representative pictures were taken at 20x and 60x magnification. Scale bars:100 μm.

**Fig 3:**
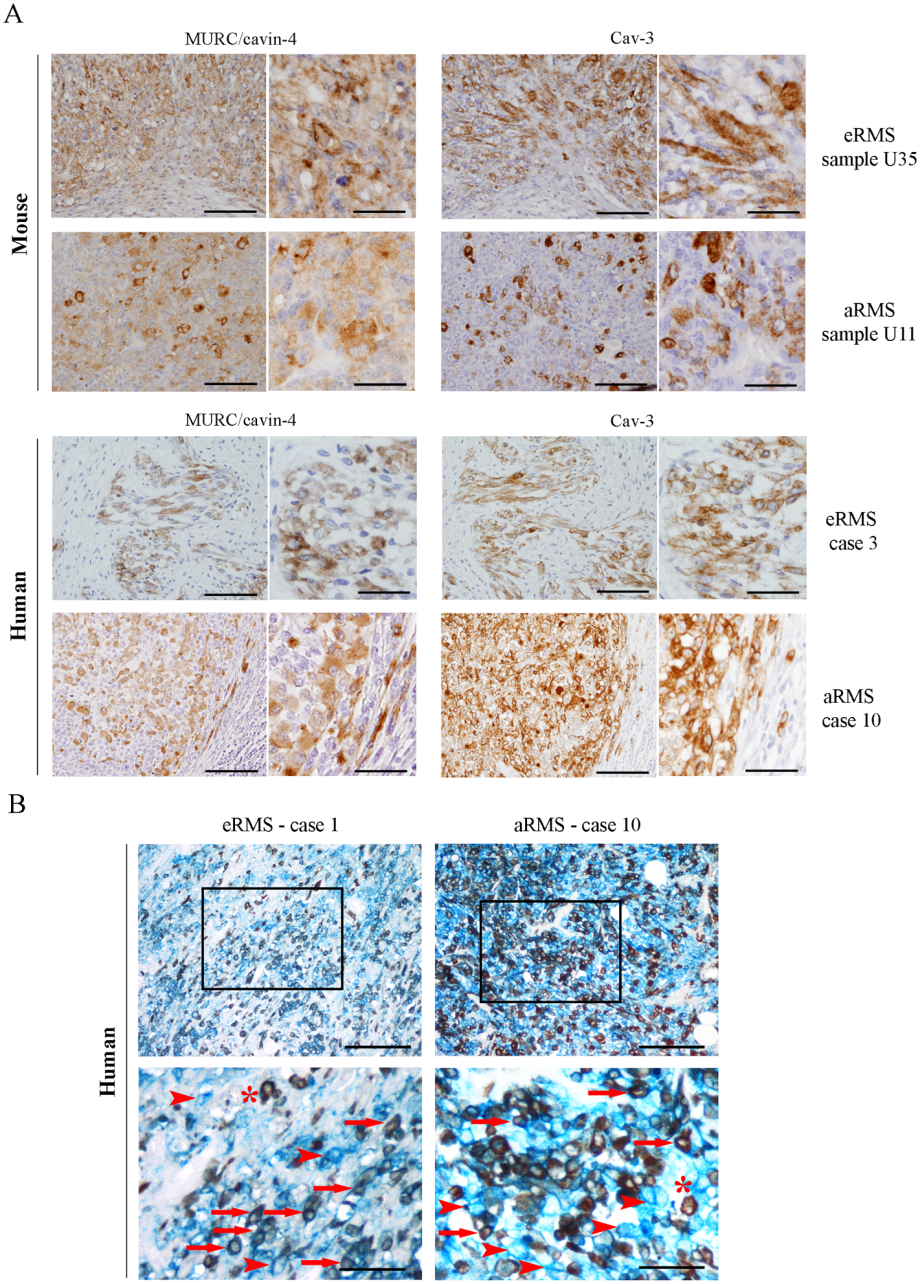
Concurrent expression of MURC/cavin-4 and Cav-3 in RMS tumors. A) Single staining (brown) of either MURC/cavin-4 or Cav-3 was evaluated by IHC analysis on serial tumor sections established from mouse and human tumor samples (as reported in Table 1). Representative pictures were taken at 20x and 60x magnification. Scale bars:100 μm. B) MURC/cavin-4 (brown) and Cav-3 (blue) staining was evaluated by double IHC analysis on human tumors. Representative pictures were taken at 20x magnification, whereas 60x magnification corresponds to inset; scale bars: 100 μm. * corresponds to MURC/cavin-4 brown staining, arrowhead corresponds to Cav-3 blue staining and arrow corresponds to double staining.

We performed an immunohistochemical (IHC) analysis using a total of 17 samples amongst mouse and human tumors (Table 1). Prior to staining tumors, we tested the specificity of MURC/cavin-4 antibody on mouse tissues, including heart, skeletal muscle and spleen; the results showed that MURC/cavin-4 staining was specifically restricted to heart and skeletal muscle (Fig 2A), and similar results were obtained on human tissues (not shown). In tumors, MURC/cavin-4 stained near the totality of samples with a proportion of cells ranging from 5% up to 90% (Table 1), as shown in representative pictures (Fig 2B). Since the observed co-expression of MURC/cavin-4 and Cav-3 in skeletal muscle [52], we investigated whether this may occur also in RMS. For this purpose, IHC analysis carried out on serial tumor sections showed that MURC/cavin-4 staining consistently overlaps that of Cav-3 in a significant number of foci/cells (Fig 3A). These findings were then confirmed by double IHC analysis carried out on human samples, where near the totality of MURC/cavin-4 expressing cells were also positive to Cav-3 (Fig 3B). Of note, we also observed a few number of Cav-3 expressing cells that were negative to MURC/cavin-4 (Fig 3B), although the majority of the cells were doubly stained for both MURC/cavin-4 and Cav-3.

**Table 1:** IHC evaluation of MURC/cavin-4 and Cav-3 expression levels in RMS tumors.

**Mouse tumor specimens**								
**Sample name**	**Histotype**	**Site of Onset**	**Morphology**	**Genetic background**	**MURC/cavin-4**		**Cav-3**	
					**% of positive cells**	**Intensity (0-1-2-3)**	**% of positive cells**	**Intensity (0-1-2-3)**
U86	eRMS	buttock	Spindle cell	Myf5Cre-PTC1^+/-^; p53^-/-^	15%	1	20%	1
U216	eRMS	back	Spindle cell	Pax7CreER-PTC1^+/-^; p53^-/-^	5%	1	10%	1
U35	eRMS	right-arm	Spindle cell	Myf5Cre-PTC1^+/-^; p53^-/-^	90%	2	90%	2
U222	aRMS	Arm	Epitheliod	Myf6Cre-Pax3^P3F/P3F^; p53^-/-^	40%	2	5%	1
U87	aRMS	Arm	Epitheliod	Myf6Cre-Pax3^P3F/P3F^; p53^-/-^	40%	1	40%	1
U11	aRMS	left-leg	Round cell neoplasm	Myf5Cre-Pax3^P3F/P3F^; p53^-/-^	40%	2 with rare elements 3	50%	2
U89	aRMS	Leg	Solid variant, epitheliod	Mcre-Pax3^P3F/P3F^; p53^-/-^	30%	2	20%	2
**Human tumor specimens**								
**Case number**	**Histotype**	**Site of Onset**	**Genetic background**		**MURC/cavin-4**		**Cav-3**	
					**% of positive cells**	**Intensity (0-1-2-3)**	**% of positive cells**	**Intensity (0-1-2-3)**
1	eRMS	Occipital	Unknown		40%	2	50%	3
2	eRMS	Nasopharinx	Unknown		5%	1	10%	1
3	eRMS	Thigh	Unknown		40%	2	60%	3
4	eRMS	Nasopharinx	Unknown		60%	2 with rare elements 3	70%	3
5	eRMS	Pelvic	Unknown		80%	2 with rare elements 3	90%	3
6	eRMS	Arm	Unknown		80%	3	90%	3
7	aRMS	Perineum	Unknown		20%	3	90%	3
8	aRMS	Thigh	Unknown		10%	2	70%	2
9	aRMS	Calf	Unknown		30%	2 with rare elements 3	80%	2
10	aRMS	Elbow	Unknown		80%	2	100%	3

Tumor samples employed for the immunohistochemical evaluation of MURC/cavin-4 and Cav-3.

Expression of Cav-3 or MURC/cavin-4 was semi-quantitatively scored on the basis of percentage of positive immunoreactive cells and staining intensity, the latter evaluated as follows: 1, low; 2, moderate; 3, strong staining intensity. Mouse tumors were established from transgenic mice with specific genetic backgrounds [54].

### *In vitro* differentiation of human and mouse RMS cultures yields a rise in the protein levels of both MURC/cavin-4 and Cav-3

**Fig 4:**
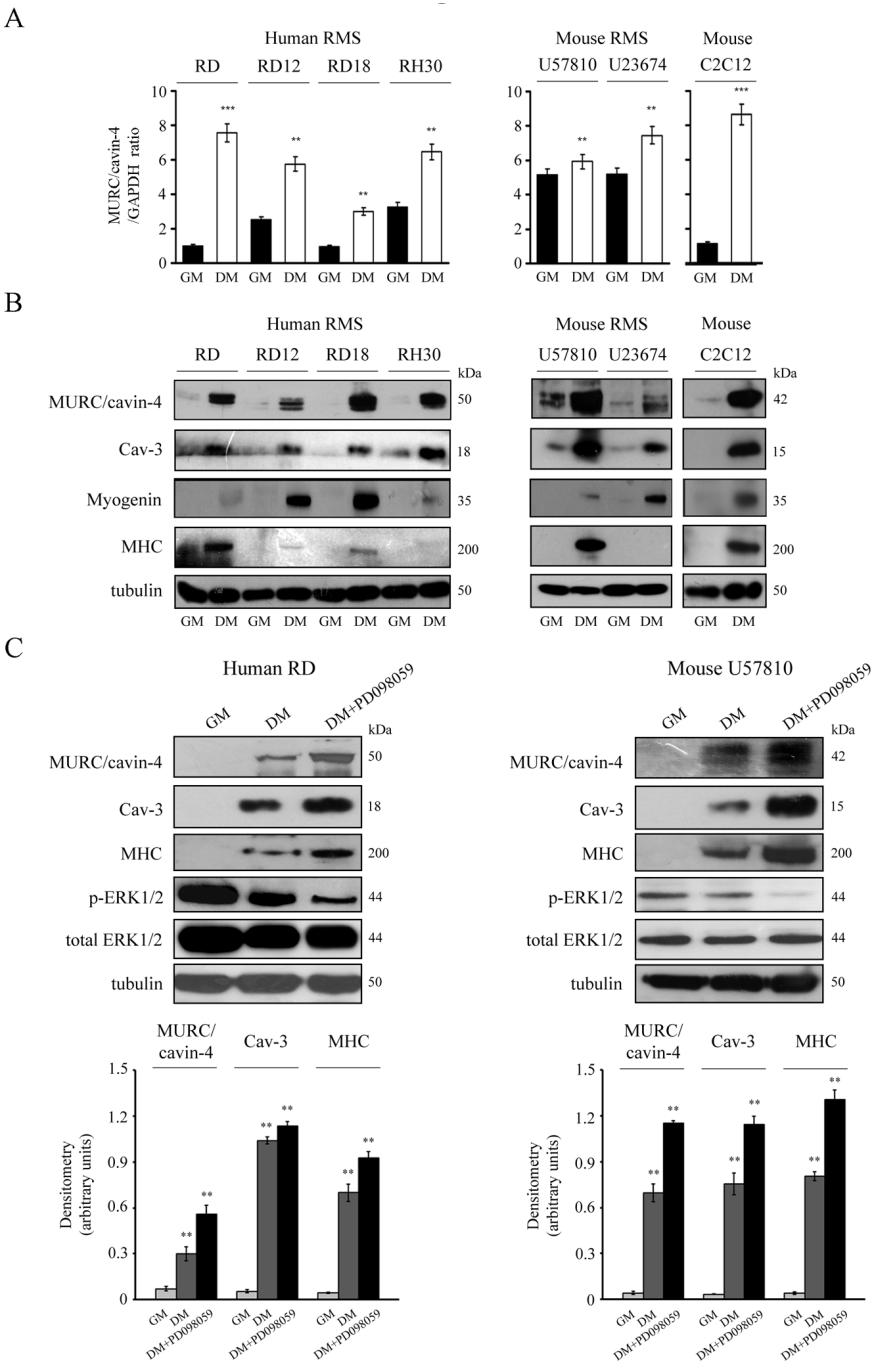
*In vitro* expression of MURC/cavin-4 and Cav-3 in RMS cultures. *In vitro* analysis of MURC/cavin-4 expression was conducted using human cell lines (embryonal RD, RD12, RD18 and alveolar RH30) and primary mouse tumor cultures (embryonal U57810 and alveolar U23674); the mouse skeletal C2C12 myoblasts served as positive control. Cells were seeded in 60-mm dishes (at a density of 12 x 10^4^) and cultured in GM until confluence, followed by incubation in DM. After 72 hours in GM or DM, cells were harvested and analyzed for transcript and protein content. A) Semi-quantitative PCR analysis was carried out to analyze the transcriptional levels of *MURC/CAVIN-4* in the different cell lines. Mean ± SD of the relative *MURC/cavin-4* levels were normalized with *GAPDH* expression; **, P < 0.001; ***, P < 0.0001. Results are representative of three independent experiments. B) Under the same conditions, immunoblotting was performed to analyze the protein content of MURC/cavin-4, Cav-3, myogenin and MHC. Results are representative of three independent experiments. C) The embryonal human RD and mouse U57810 cells were seeded in 60-mm dishes (at a density of 12 x 10^4^) and maintained in GM for up to 72 hours. Cells were then differentiated in the presence of DM or DM added with 10 μM PD098059 for additional 72 hours. The derived cell homogenates were then used to analyze the protein content of MURC/cavin-4, Cav-3, MHC, ERK1/2 (both phosphorylated and total forms) by immunoblotting. Protein bands were quantified by densitometry after normalization with respect to tubulin (n = 3). **, P < 0.001.

An *in vitro* investigation was carried out using human cell lines (embryonal RD, RD12 and RD18 or alveolar RH30) and mouse primary tumor cultures (embryonal U57810 and alveolar U23674). As detected via semi-quantitative RT-PCR analysis, the transcriptional *MURC/CAVIN-4* levels were consistently increased in all the cell lines cultured in differentiating medium (DM) as compared to growth medium (GM) (Fig 4A). This evidence was confirmed by immunoblotting analysis showing that the MURC/cavin-4 protein levels were low or almost undetectable in all the proliferating cell lines but incremented concurrently with Cav-3 and myogenin during myogenic differentiation (Fig 4B); in addition, increased levels of MHC, a marker of terminal differentiation, were only observed in eRMS lines, being aRMS lines usually more refractory to complete the differentiation process [53, 54] (Fig 4B). Of note, in human and mouse lines we estimated the molecular weight of MURC/cavin-4 to be approximately 50 and 42 kDa, respectively (Fig 3B). To further strengthen our findings, we also forced the differentiation by co-treating the human RD and mouse U57810 cells with DM and an upstream inhibitor of the ERK1/2 phosphorylation, namely PD098059 [57, 59]. This treatment was indeed effective to reduce the levels of phosphorylated ERK1/2 levels in comparison to DM and GM conditions, leading to improved morphological cell differentiation (not shown), which was characterized by a robust increase of MURC/cavin-4, Cav-3 and MHC protein levels, as detected by immunoblotting (Fig 4C). Consistent with this, the same treatment performed on aRMS lines, having no effect on the extent of myogenic differentiation, did not change the protein levels of MURC/cavin-4 and Cav-3 (not shown).

Taken together, the *in vitro* data suggest that MURC/cavin-4 and Cav-3 protein levels concurrently increase during differentiation of RMS cells.

### During myogenic differentiation MURC/cavin-4 and Cav-3 colocalize at the plasma membrane in human RD and RH30 cells

**Fig 5:**
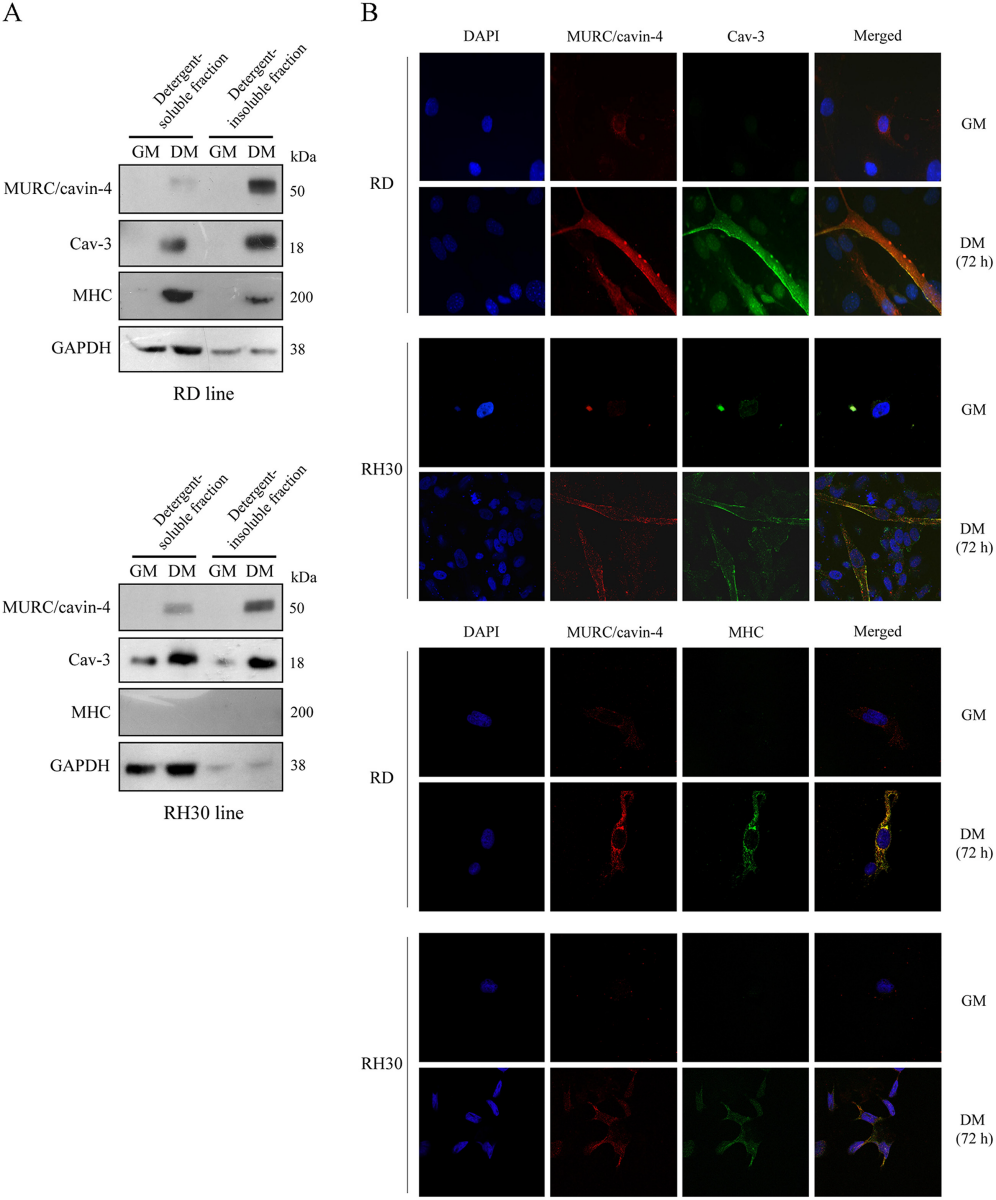
Subcellular localization of MURC/cavin-4 and Cav-3 in the human RD and RH30 lines. A) Embryonal RD and alveolar RH30 cells were seeded in 60-mm dishes (at a density of 12 x 10^4^) and cultured in GM for 72 hours until reaching confluence; cells were then treated with DM for additional 72 hours. Protein homogenates were subjected to cell fractionation, and the detergent-soluble and-insoluble fractions were analysed by immunoblotting to evaluate the protein levels of MURC/cavin-4, Cav-3, MHC and GAPDH. Results are representative of three independent experiments. B) Confocal microscopy analysis was employed to analyze the distribution of MURC/cavin-4 (red), Cav-3 (green) and MHC (green) in RD and RH30 cells cultured in GM or DM. Nuclei were counterstained with DAPI (blue). Samples were analyzed using a Zeiss LSM510 META microscope equipped with a 63x oil immersion objective. Merged images, captured using the LSM 510 Meta software, showed an extensive co-localization of MURC/cavin-4 with Cav-3 at the cell surface as well as with MHC in the cytosol (yellow signal). Pictures were taken at 63x magnification.

We investigated the cellular distribution of MURC/cavin-4, Cav-3 and MHC using the human embryonal RD and alveolar RH30 lines. By means of cell fractionation followed by immunoblotting, we observed MURC/cavin-4 protein levels to be concurrently increased with Cav-3 during cell differentiation and mainly enriched in the detergent-insoluble cell fractions (Fig 5A). In differentiated cell lines Cav-3 was found to be enriched in both cell fractions (Fig 5A), whereas MHC was mostly detected in detergent-soluble fractions only in differentiated RD cells (Fig 5A). Immunofluorescence analysis showed that MURC/cavin-4 staining co-localized with that of Cav-3 at the cell surface in differentiated RD and RH30 lines, while their labelling being hardly detectable in proliferation (Fig 5B, top panels). We also observed a strong co-staining of MURC/cavin-4 and MHC in differentiated RD cells (Fig 5B, bottom panels), while a weaker co-staining was visualized in a few number of likely more differentiated RH30 cells (Fig 5B, bottom panel), despite the lack of MHC detection by immunoblotting (Fig 5A). Overall, these data indicate that MURC/cavin-4 and Cav-3 co-localize at the plasma membrane in more differentiated RD cells (positive to MHC) as well as in less differentiated RH30 cells (negative to MHC).

### MURC/cavin-4 silencing impairs differentiation of the embryonal RD cell line

**Fig 6:**
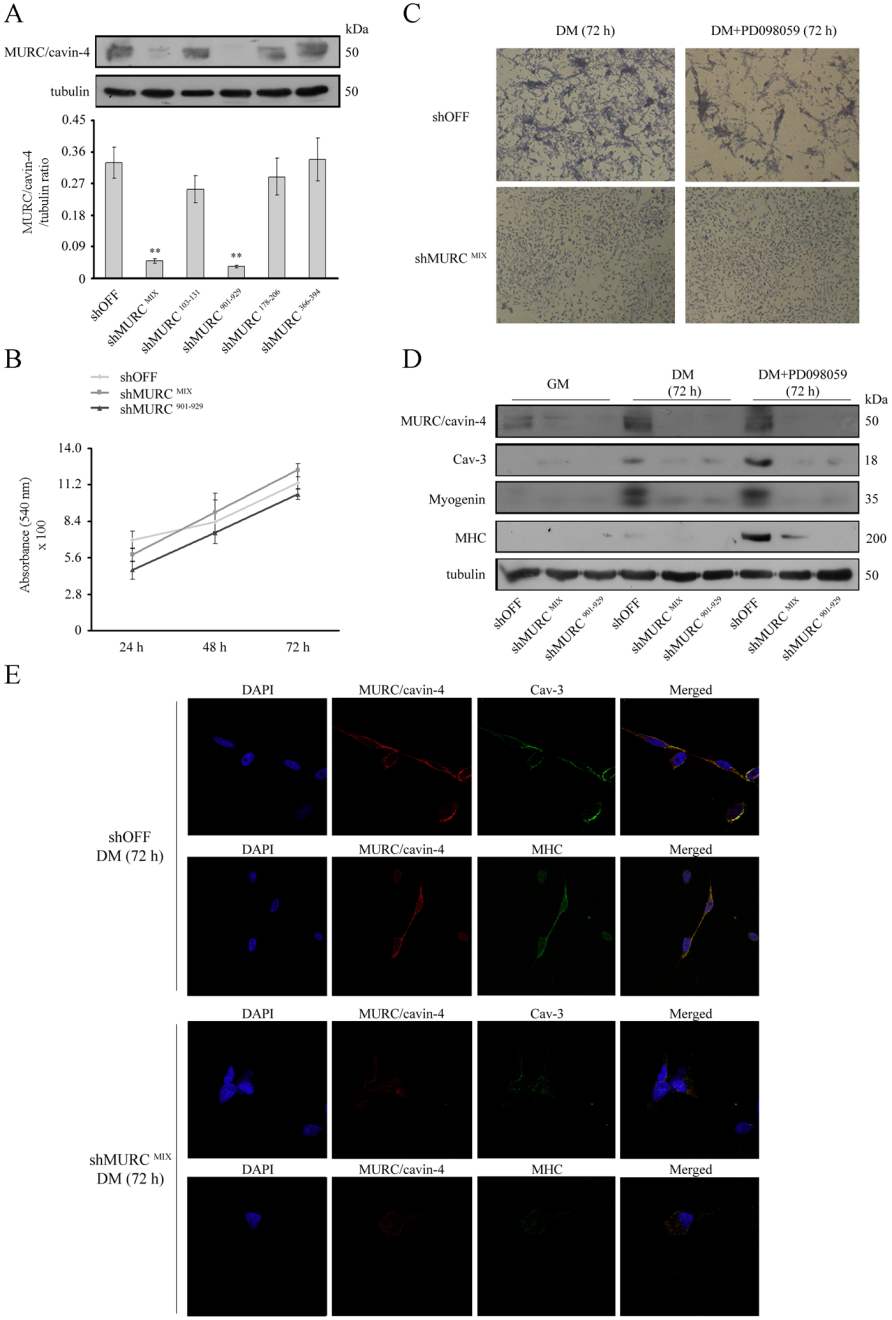
Effects of MURC/cavin-4 knockdown in the RD cell line. Stably transfected RD clones (i.e., control shOFF and five different knock-down clones, namely shMURC ^MIX; 103–131; 901–929; 178–206; 366–394^) were seeded in 60-mm dishes (at a density of 12 x 10^4^) and harvested after 72 hours in GM. SDS-PAGE was carried out loading gel with 160 μg proteins per each sample and immunoblotting was performed to evaluate the protein levels of MURC/cavin-4. Results are representative of three independent experiments. Protein bands were quantified by densitometry after normalization with respect to tubulin (n = 3). **, P < 0.001. B) Crystal violet assay was employed to compare the proliferation over a time-course of 24-48-72 hours in knock-down MURC/cavin-4 clones (i.e., shMURC ^MIX^ and shMURC ^901–929^) and control shOFF clone. Results are representative of three independent experiments. C) Control shOFF and knock-down shMURC ^MIX^ cells were seeded in 60-mm dishes (at a density of 12 x 10^4^) and, once reached the confluence, were differentiated in the presence of DM or DM added with 10 μM PD09859 for 72 hours. Giemsa staining was then employed to visualize the morphological differentiation. Pictures were taken under a phase contrast microscope at 40x magnification. Images are representative of three independent experiments. D) Under the same conditions, immunoblotting was performed to evaluate the protein content of MURC/cavin-4, Cav-3, myogenin and MHC. Results are representative of three independent experiments. E) Confocal microscopy analysis was employed to analyse the distribution of MURC/cavin-4 (red), Cav-3 (green) and MHC (green) in knock-down shMURC ^MIX^ clone as compared to control shOFF clone cultured in DM. Nuclei were counterstained with DAPI (blue). Samples were analyzed using a Zeiss LSM510 META microscope and pictures were taken with a 63x oil immersion objective.

We investigated whether silencing MURC/cavin-4 by shRNAi may affect the myogenic differentiation. For this purpose, the RD cells were transfected either with four different shMURC vectors (shMURC ^103–131; 901–929; 178–206; 366–394^) or with a mixture of all (shMURC ^MIX^), using a random OFF-target sequence as a negative control (shOFF). To assess the levels of MURC/cavin-4 knock-down in stably transfected clones, immunoblotting analysis was performed loading double the amount of protein samples (160 μg) and using heavy exposures, since the low MURC/cavin-4 expression detected during cell proliferation. The analysis on five independent clones showed a significant down-regulation of MURC/cavin-4 levels in shMURC ^MIX^ and shMURC ^901–929^ clones as compared to control shOFF cells (Fig 6A). Similar results were also obtained by analysis of *MURC/CAVIN-4* transcript levels (not shown). We then decided to evaluate whether MURC/cavin-4 knockdown may affect the cell cycle. As measured by means of crystal violet assay, we found that proliferation of shMURC ^MIX^ and shMURC ^901–929^ cells was similar to that of control shOFF cells over a time-course of 72 hours (Fig 6B).

The effects of MURC/cavin-4 silencing on differentiation were then evaluated by staining cells with Giemsa reactive. While culturing the clones in the presence of DM or DM added with 10 μM PD09859 for up to 72 hours lead shOFF cells to acquire an elongated morphology that is characteristic of differentiation, shMURC ^MIX^ cells maintained a round-shape morphology (similar results were observed in shMURC ^901–929^ cells, not shown) (Fig 6C). Under these conditions, immunoblotting analysis showed that the increased and concurrent expression of MURC/cavin-4, Cav-3, myogenin and MHC, as observed in shOFF cells, was severely abrogated in both shMURC clones (Fig 6D). In addition, immunofluorescence analysis showed that the co-staining of MURC/cavin-4 with Cav-3 or MHC, as observed in differentiated shOFF cells, was almost completely depleted in shMURC ^MIX^ cells (Fig 6E), and similar results were observed in shMURC ^901–929^ cells (not shown).

## Discussion

MURC/cavin-4 and Cav-3 are protein members of the Cavin [38–43] and Caveolin [36, 37] families, essential coat components and regulators of caveola biogenesis [44, 45]. Both these proteins share a restricted tissue-specific expression in cardiac and skeletal muscle tissues, where they play different non redundant but sometimes overlapping roles. In striated muscle MURC/cavin-4 was found to be associated to the Z-line [51], the structural border of the sarcomere which also serves as a platform for a large number of the Z-disc-associated proteins that shuttle between the Z-disc and other subcellular locations to transmit signals [60]. *MURC/CAVIN-4* gene knock-down or over-expression has been shown to impair or improve the differentiation of mouse C2C12 myoblasts through decreased or increased ERK1/2 activation in the later stages of differentiation [51]. Cav-3 is a membrane scaffolding protein [61–63] that interacting at the sarcolemma with a number of signalling proteins, such as nitric oxide synthase [64, 65], TGF-β (transforming growth factor-beta) receptors [66, 67] and dysferlin [68, 69], is involved in the regulation of many processes, including skeletal muscle differentiation and regeneration. Mutated Cav-3 forms affect the survival and differentiation of myoblasts [70, 71] and are involved in the onset of cardiac and neuromuscular disorders [72–75], such as the Limb Girdle Muscular Dystrophy 1-C [64, 65, 76, 77]. Furthermore, Cav-3 deficient muscles from dystrophic patients display a loss of MURC/cavin-4 [52], suggesting that MURC/cavin-4 and Cav-3 may together cooperate for proper functioning of skeletal muscle tissue.

RMS are pediatric tumors mainly deriving from myogenic lineages [53, 54, 78–80] and showing distinctive traits found in skeletal muscle, including the appearance in the cytoplasm of the typical striated bands corresponding to sarcomere structures and expression of muscle-specific markers, such as MyoD, myogenin, muscle specific actin (MSA), desmin, sarcomeric alpha-actin, and myoglobin. RMS cells carry genetic alterations that hinder the cell cycle withdrawal and/or prevent the myogenic differentiation process [7]. Poorly differentiated RMS cells have higher probability to metastasize [81], and therefore increasing their differentiation potential could, in principle, irreversibly arrest cell proliferation to control the disease with less side effects than conventional therapies [82]. In this context, the translational research of markers helping to predict the status of RMS cell differentiation is precious.

In 2005, a study reported Cav-3 to be expressed in the more differentiated eRMS and aRMS subsets, thereby configuring this protein as a useful marker to assess the degree of differentiation or detect residual tumor cells that may undergo differentiation following chemotherapy [49]. Afterwards, a study from our group has corroborated these findings showing that Cav-3 expressing cells are often positive for MHC, a marker of terminal differentiation, and further suggesting that Cav-1, which is a marker of muscle satellite cells highly homolog to Cav-3, instead configures as a marker of poor differentiation in RMS [46]. In the present study we showed MURC/cavin-4 and Cav-3 to be frequently co-expressed in human RMS specimens as well as in mouse tumors established in transgenic mouse models that authentically recapitulate the onset and progression of eRMS and aRMS subsets [54]. Our *in silico* analysis revealed that MURC/cavin-4, Cav-3 and MHC had all a similar trend of expression, being less to more expressed in aRMS, eRMS and skeletal muscle, respectively. In addition, we found a significant correlation between MURC/cavin-4 and Cav-3 among tumor samples and also detected an increased survival’s probability for RMS patients with higher Cav-3 expression, indicating that Cav-3 signature may be associated to a better prognosis, likely due a major degree of tumor differentiation. In light of this evidence, we may hypothesize that MURC/cavin-4 may have a similar behavior, although this still awaits further investigation. The observed *in silico* correlation between MURC/cavin-4 and Cav-3 was found to occur also *in vivo* and *in vitro*; indeed, immunohistochemical analysis of tumor samples detected MURC/cavin-4 staining frequently in Cav-3 expressing cells, whereas a robust and concurrent expression of both MURC/cavin-4 and Cav-3 was observed in human RMS lines and mouse primary tumor cultures only during differentiation. Interestingly, whereas near all the MURC/cavin-4 expressing RMS cells were positive to Cav-3, we also recognized a number of cells positive for Cav-3 and negative for MURC/cavin-4. This could indicate that Cav-3 expression during the differentiation process may occur prior to MURC/cavin-4 expression. In this regard, we must also carefully taken into account that both eRMS and aRMS tumors can frequently express myogenic markers of every cell stage irrespective of a real differentiation program [54]. Although MURC/cavin-4 and Cav-3 levels increased upon differentiation stimuli, this up-regulation was strongly associated with terminal differentiation due to MHC expression only in cell lines representative of eRMS, which are known to exhibit the greatest extent of myodifferentiation [54]. Instead, in the less prone differentiating and more aggressive aRMS tumors [54] we noted an increased expression of MURC/cavin-4 and Cav-3 but lack or very low expression of MHC; this means that MURC/cavin-4 and Cav-3 co-expression cannot be univocally interpreted as readout of terminal differentiation, as they may even feature an early/intermediate stage of differentiation. Likely, MURC/cavin-4 or Cav-3 expression alone is not sufficient to efficiently predict the status of myogenic differentiation in RMS cells. MURC/cavin-4 resemble myogenin: RMS are highly positive for this marker, that normally increases during myogenesis, when they are enforced to differentiate. However, myogenin itself is not predictive of the status of RMS differentiation.

In differentiating RMS cells we observed both proteins residing in the cytosol but mainly co-localizing at the plasma membrane, indicating that their activity may underlie the proper extent of differentiation in RMS, as occurring in skeletal muscle [51, 52]. Consistent with this, *MURC/CAVIN-4* gene silencing was sufficient to impair the differentiation process in the human RD cell line, leading to loss of expression of myogenin, Cav-3 and MHC. These findings strengthen the importance of MURC/cavin-4 as a readout of differentiation in RMS, and further opens new interesting questions on the MURC/cavin-4-dependent mechanisms underlying RMS differentiation, which will be the matter of future investigation. In this regard, MURC/cavin-4 was shown to influence differentiation in skeletal muscle cells by ERK pathway activation [51], whereas to modulate cardiac function by activating the Rho—ROCK (RAS homolog/Rho-associated protein kinase) pathway and recruiting ERK to caveolae in response to adrenergic stimulation [83][84]. In conclusion, this work points out that MURC/cavin-4, likely cooperating with Cav-3, is required for RMS cells to undergo myogenic differentiation.
